# Supplementary material for: Unsupervised monocular depth estimation with omnidirectional camera for 3D reconstruction of grape berries in the wild
Source: PLoS One. 2025 Feb 3;20(2):e0317359. doi: 10.1371/journal.pone.0317359 (PMC11790092; doi:10.1371/journal.pone.0317359)
Supplement: Attachment — Submitted filename: reply_letter.pdf [file pone.0317359.s002.pdf]

Response to the reviewers

We thank the reviewers for their critical assessment of our work. In the following, we address their concerns point by point. Newly added or revised sections are indicated in red in the revised manuscript.

Reviewer 1

Reviewer Point P 1.1 — The structure of the paper should seriously be improved:

a. The introduction Section is not written to present the importance of the solved problem. I was confused. What is the main problem /s that you have solved? Please reduce the title of paper length.

b. The related works should be discussed and the prons and coins should be expressed in one paragraph in the introduction Section to make the proposed method/s's efficient.In related work section, please add new references that are published in state-of-the-art. In addition. The logic of the mentioned references needs to be strengthened to highlight the innovations of this paper. The difficulty encountered in this research should be discussed in more detail. Please compare and add this references:

- Car depth estimation within a monocular image using a light CNN
- A New Approach To Estimate Depth Of Cars Using A Monocular Image
- An Efficient Approach to Monocular Depth Estimation for Autonomous Vehicle Perception Systems

Reply: Thank you for pointing out that the problems solved in the paper were difficult to understand. This paper addresses the challenge of estimating the depth of grape bunches from omnidirectional camera images to facilitate their 3D reconstruction. We have revised the Introduction section to make the problems easier to understand. We have also shortened the title.

We have already cited the representative monocular depth estimation methods in the previous manuscript. In the papers suggested by the reviewer, experiments were conducted applying the methods to datasets captured by an in-vehicle camera. Therefore, although the reviewer suggested to cite these papers, we have decided not to do so because citing the papers do not improve the value of our paper.

Reviewer Point P 1.2 — The paper should be provided some realistic scenario with noises... to access the performanceof proposed approach.

Reply: Thank you for your comments on the experiment. The experiment was conducted in a field for table grapes, which require thinning. As explained in Fig. 2 and the 3rd paragraph of the Introduction section, the grape trees used in this study is trellis-shaped, not hedge-shaped, and the grape bunches hang down from the trellis. Because this trellis-type grape cultivation method is common in Japan for growing table grapes, the movies like those used in the experiment can be taken in ordinary grape fields. Therefore, this research has been sufficiently evaluated for its performance in a realistic scenario. To clarify this, we have added some explanation on 445–449 of the revised manuscript.

Reviewer Point P 1.3 — Overall, the paper is written in good English and style. However, there are some typos as well as grammatical errors. Could the authors please revise and proof-read the paper carefully before resubmission for publication?

Reply: Thank you for pointing this out. We have carefully read the manuscript and fixed mistakes before re-submission.

Reviewer 2

Reviewer Point P 2.1 — It would be better to add the recorded field video either in MS or Supplementary for the readers.

Reply: Thanks for your useful comment. We've added the video dataset to supporting information.

Reviewer Point P 2.2 — Some of the abbreviations have been used in the MS, which need to be written as a full name at the beginning and later can be used in the abbreviated forms.

Reply: Thank you for bringing this to our attention. Upon reviewing the manuscript, we realized that the full name of RGB was missing, and we have now included it.

Reviewer Point P 2.3 — Add some more detail to the discussion sections, please.

Reply: We appreciate the reviewer's comment. However, we were unable to clearly identify the specific aspect the reviewer intended to highlight. After carefully reviewing the discussion section, we believe that the current content is appropriate as it stands. Therefore, we have not made any changes to this section. If the reviewer has further specific suggestions or concerns, we would be happy to address them.

Minor

Reviewer Point P 2.4 — Abstract. Write "arrangements" instead of "arrangement".

Reply: Thank you for pointing that out. We fixed it.

Reviewer Point P 2.5 — Abstract. rewrite "labor-intensive" instead of "labor intensive".

Reply: Thank you for your comment. We fixed it in the abstract and other part of the manuscript.

Reviewer Point P 2.6 — Lines 1-3, kindly provide reference/source.

Reply: Thank you for your point. We've added two references.

Reviewer Point P 2.7 — Lines 4-6, kindly provide reference/source.

Reply: Thank you for your point. We've referred to the paper added in P 2.6. In addition, as there was no appropriate article that showed the price for a bunch of grapes, we have referred to a web article

that described grapes that were auctioned off at a high price and the reasons why grapes are traded at high prices in Japan and rewrote some sentences.

Reviewer Point P 2.8 — Figures 2 and 3 are repetitions of the same phenomena (stereo vision), which need to be presented in a single figure having subsections a, b, and c.

Reply: Thank you for your comment. We have fixed it according to the comment.

Reviewer Point P 2.9 — Line 243, 360 and 374 "fig. 5(B).", fig. 7 (A) and fig. 8 (A) need to be "Fig. 5(B)", "Fig. 7 (A)" and Fig. 8 (A) respectively.

Reply: Thank you for your point. We've fixed them.

Reviewer 3

Reviewer Point P 3.1 — 1. No keywords.

Reply: We checked the submission guidelines for PLOS ONE, but there was no rule about including keywords. Since there is no rule about keywords in the submission guidelines, we will not include them in the main text. We filled keywords in the system when we resubmitted the manuscript. Just to be sure, here are the keywords for this paper:
table grape cultivation, pruning/thinning, agricultural automation, computer vision, RGB-D perception, deep learning.

Reviewer Point P 3.2 — I suggest change the title of figure 1 to (Proposed grape pruning support system).

Reply: Thank you for your point. We've fixed it according to the comment.

Reviewer Point P 3.3 — The paper lacks of literature reviews.

Reply: Thank you for your comment. We've received similar comments in P 2.6 and and added some references. Please find P 2.6 and P 2.7.

Reviewer 4

Reviewer Point P 4.1 — The manuscript lacks a detailed description of the neural network architecture used for depth and pose estimation. For instance, it does not specify the number of convolutional layers, kernel sizes, activation functions, or the overall architecture of the encoder-decoder framework. Providing this information is crucial for enabling replication and deeper understanding of the proposed method by the research community.

Reply: Thank you for highlighting this issue. We used the same network architectures for both the depth and pose networks as [16]. We have added a new section specifically dedicated to describing these architectures.

Reviewer Point P 4.2 — While the proposed method is effective under certain conditions, the manuscript could benefit from a more thorough discussion of how varying environmental factors, such as changes in lighting conditions and the different growth stages of grape berries, might affect the performance of the system. These factors are critical in agricultural applications, where conditions can be highly variable.

Reply: Thank you for your insightful comments. We believe that the differences in growth stages, weather, and field conditions can be addressed through additional training. A discussion on this has been added to lines 452–459 of the revised manuscript.

Reviewer Point P 4.3 — It would be valuable for the authors to clarify whether the primary objective of this research is to assist human workers by providing a consistent standard for grape pruning, or if it is intended as a preparatory step toward full automation of the pruning process. Each objective might require different approaches in terms of system design and evaluation criteria.

Reply: Thank you for your comment. Lines 24—26 of the Introduction explains that the primary objective of this research is to support human workers by establishing a consistent standard for grape pruning.

Reviewer Point P 4.4 — Conduct more real environment tests and present these results in the paper. This should include realistic scenarios for testing system performance under various conditions, highlighting its strengths and areas for improvement.

Reply: Thank you for your thoughtful comment. As outlined in the manuscript, the experiments were conducted using real data collected from actual grape fields. Therefore, we believe that the concerns raised by the reviewer have already been addressed in the original manuscript. The above content has been added to lines 431–435 of the revised manuscript.

Reviewer 5

Reviewer Point P 5.1 — The manuscript presents technical and scientific merits about: Unsupervised Monocular Depth Estimation with Omnidirectional Camera and Its Application to Obtaining 3D Structure of Textureless, Symmetric and Crowded Grape Berries in the Wild. The Manuscript falls within the scope of the Plos One. However, from my expertis it is necessary to detail certain points so that the audience has a better understanding.

Reply: Thank you for your comment. We have revised the paper, taking into account the comments from other reviewers as well.

Genral comment

Reviewer Point P 6.1 — From lines 2 to 9, supporting it with scientific evidence is suggested.

Reply: Please see P 2.6.

Reviewer Point P 6.2 — From lines 12 to 23, it is suggested to support it with scientific evidence.

Reply: We added 3 references.

Reviewer Point P 6.3 — Same comment for line 24 to 34.

Reply: This suggestion is a little ambiguous because it is not sufficiently clear to us which point the reviewer means. However, we guess the point is "Unlike wine grape cultivation in vineyards, with Japanese table grape cultivation, farmers need to work in confined spaces where grape bunches hang from trellises at a height of about 1.6 meters." To show scientific evidence, what we can is to show photos of Japanese grape wine fields. Therefore, we added a new figure in Fig. 1 of the revised manuscript.

Reviewer Point P 6.4 — On line 59 it is suggested to specify which research subject has been used.

Reply: We used monocular depth estimation to acquire the 3D shape of grape bunches.

Reviewer Point P 6.5 — Also on line 69-70, support with scientific evidence.

Reply: We couldn't find the article that stated that the majority of monocular depth estimation is for perspective projection images. Therefore, we added the phrase "as far as we know" to the sentence.

Reviewer Point P 6.6 — It is suggested to add the scientific name of the grape.

Reply: Scientific names of the grapes we used were as follows: 'Fujiminori' grape (*Vitis vinifera* L. × *V. labruscana* Baily), 'Kyoho' grape (*Vitis vinifera* L. × *V. labruscana* Baily), 'Sun Verde' grape (*Vitis labruscana* Baily × *V. vinifera* L.) and 'Shine Muscat' grape (*Vitis labruscana* Baily × *V. vinifera* L.). We have added the names in the revised manuscript.

Reviewer Point P 6.7 — It is suggested not to clutter the composite Figure 1 with text. I suggest using letters A, B, C, etc. And in the bottom of the figure, make the description.

Reply: Thank you for your suggestion to improve the figure. We have edited Fig. 1, added figure numbers (a), (b), and (c) to each panel, and added explanations to the caption. Due to the addition of a new figure, it has been changed to Fig. 2. Please check the revised Fig. 2.

Reviewer Point P 6.8 — In Figure 1, what do the red circles represent?

Reply: Red circles represent the berries that are judged to be cut off.

Reviewer Point P 6.9 — Figure 2 is not mentioned in the body of the Introduction.

Reply: We have moved the figure to **Materials and Methods**.

Reviewer Point P 6.10 — It is suggested to add the objective of the research and its hypothesis.

Reply: We have rewritten **Introduction** to clarify the objective of the research.

Reviewer Point P 6.11 — It is suggested to add the permissions you obtained to carry out the research work.

Reply: We did not need to obtain any kind of permission to carry out this experiment, so we did not obtain any.

Reviewer Point P 6.12 — In line 122, reference is made to Figure 2, so it is suggested to place it immediately after its mention. Same comment for the other Figures.

Reply: Thank you for your suggestion. We have revised the manuscript accordingly.

Reviewer Point P 6.13 — Indicate in which phenological stage of the crop the images were read.

Reply: We captured the images of bunches on the fruit set stage (E-L system 31–32). We have added this in line 121 of the revised manuscript.

Reviewer Point P 6.14 — On line 132, do not start the sentence with abbreviations.

Reply: We made the correction as you indicated.

Reviewer Point P 6.15 — It is suggested to arrange Figure 7 horizontally. Furthermore, the letters A, B are not observed as indicated in the figure caption.

Reply: Thank you for your suggestion. We modified the manuscript accordingly. Furthermore, we have added letters (a) and (b) to the figure.

Reviewer Point P 6.16 — On line 374 it is repeated fig. 8. fig. 8 (A). Check in the authors guide whether it is written in lowercase or uppercase (fig. or Fig.)

Reply: This is not a repeat. The first sentence ends with fig. 8., and the next sentence begins with fig. 8 (A). However, as you pointed out in P 2.9, we must not start a sentence with an abbreviation, so we have corrected the second fig. 8 (a) to Figure 8 (a).

Reviewer Point P 6.17 — In Figure 8, specify what the X, Y and Z axis corresponds to.

Reply: X, Y, and Z represent the three orthogonal coordinates in three-dimensional space when an arbitrary point is used as the origin. There are no units.

Reviewer Point P 6.18 — On line 343: The frame size of the videos used for the experiment was 480×480 px, and the frame rate was 30 fps. It is suggested not to abbreviate the unit of measurement.

Reply: Thank you for pointing this out. px is pixels, and fps is frames per second. We have corrected these to complete expressions.

Reviewer Point P 6.19 — Line 353 will be described in detail in materials and methods.

Reply: The sentence you pointed out has been moved to lines 307–308 in **Materials and Methods.**

Reviewer Point P 6.20 — For a better understanding of this section, it is suggested to discuss based on the subtitles of the Results.

Reply: We have rewritten the discussion section, as you pointed out.

Reviewer Point P 6.21 — It is suggested to incorporate the limitations and future works derived from this research.

Reply: We have already written about the limitations of the method and future work in previous manuscripts, but we have rewritten them to understand them easily. Please check lines 436–451 of the revised version.

Reviewer Point P 6.22 — It is suggested to add whether this technology is economically profitable for the grape producer.

Reply: Thank you for your suggestion. If this technology is developed, even people who have never grown grapes before will be able to help with the cultivation. This will make it possible to increase the number of workers more easily than before, leading to an expansion of cultivation. It can also be said that this technology will lead to the automation of cultivation work, and it is thought that it will lead to a reduction in the cost of grape cultivation. The above has been added to lines 460–471 of the revised manuscript.
