# Supplementary material for: Unsupervised monocular depth estimation with omnidirectional camera for 3D reconstruction of grape berries in the wild
Source: PLoS One. 2025 Feb 3;20(2):e0317359. doi: 10.1371/journal.pone.0317359 (PMC11790092; doi:10.1371/journal.pone.0317359)
Supplement: Attachment — Submitted filename: comments to manuscript_PONE-D-24-25787.docx [file pone.0317359.s001.docx]

General comments

The manuscript presents technical and scientific merits about: Unsupervised Monocular Depth Estimation with Omnidirectional Camera and Its Application to Obtaining 3D Structure of Textureless, Symmetric and Crowded Grape Berries in the Wild. The Manuscript falls within the scope of the Plos One. However, from my expertis it is necessary to detail certain points so that the audience has a better understanding.

It is suggested that the usefulness of the developed technology be made clear throughout the document, since sometimes it refers to pruning or cutting grape fruits. Both terms are different.

The Discussion section must be supported by more scientific evidence.

Introduction

From lines 2 to 9, it is suggested to support it with scientific evidence.

From lines 12 to 23, it is suggested to support it with scientific evidence.

Same comment for line 24 to 34.

On line 59 it is suggested to specify which research subject has been used.

Also on line 69-70, support with scientific evidence.

It is suggested to add the scientific name of the grape

It is suggested not to clutter the composite Figure 1 with text. I suggest using letters A, B, C, etc. And in the bottom of the figure, make the description.

In Figure 1, what do the red circles represent?

Figure 2 is not mentioned in the body of the Introduction.

It is suggested to add the objective of the research and its hypothesis.

Materials and methods

It is suggested to add the permissions you obtained to carry out the research work.

In line 122, reference is made to Figure 2, so it is suggested to place it immediately after its mention. Same comment for the other Figures.

Indicate in which phenological stage of the crop the images were read.

On line 132, do not start the sentence with abbreviations.

It is suggested to arrange Figure 7 horizontally. Furthermore, the letters A, B are not observed as indicated in the figure caption.

Results

On line 374 it is repeated fig. 8. fig. 8 (A). Check in the authors guide whether it is written in lowercase or uppercase (fig. or Fig.)

In Figure 8, specify what the X, Y and Z axis corresponds to.

On line 343: The frame size of the videos used for the experiment was 480 × 480 px, and the frame rate was 30 fps. It is suggested not to abbreviate the unit of measurement.

Line 353 will be described in detail in materials and methods.

Discussion

For a better understanding of this section, it is suggested to discuss based on the subtitles of the Results.

It is suggested to incorporate the limitations and future works derived from this research.

It is suggested to add whether this technology is economically profitable for the grape producer.

In the end I hope that these suggestions improve the structure of the manuscript, and have a better impact on readers.
